# Graves’ orbitopathy as a rare disease in Europe: a European Group on Graves’ Orbitopathy (EUGOGO) position statement

**DOI:** 10.1186/s13023-017-0625-1

**Published:** 2017-04-20

**Authors:** P. Perros, L. Hegedüs, L. Bartalena, C. Marcocci, G. J. Kahaly, L. Baldeschi, M. Salvi, J. H. Lazarus, A. Eckstein, S. Pitz, K. Boboridis, P. Anagnostis, G. Ayvaz, A. Boschi, T. H. Brix, N. Currò, O. Konuk, M. Marinò, A. L. Mitchell, B. Stankovic, F. B. Törüner, G. von Arx, M. Zarković, W. M. Wiersinga

**Affiliations:** 1 Institute of Genetic Medicine, Newcastle University, Central Parkway, Newcastle upon Tyne, Tyne, NE1 3BZ UK; 2 Department of Endocrinology and Metabolism, Odense University Hospital, Odense, 5000 Denmark; 3 Endocrine Unit, University of Insubria, Ospedale di Circolo, Viale Borri, 57 21100 Varese, Italy; 4 Department of Clinical and Experimental Medicine, Section of Endocrinology, University of Pisa, Via Paradisa 2, 56124 Pisa, Italy; 5 Department of Medicine I, Johannes Gutenberg University Medical Center, Mainz, 55101 Germany; 6 Department of Ophthalmology, Université Catholique de Louvain, Cliniques Universitaires Saint-Luc, Brussels, Belgium; 7 Graves’ Orbitopathy Center, Endocrinology, Fondazione Ca’ Granda IRCCS, Department of Medical and Community Sciences, University of Milan, Milan, Italy; 8 Institute of Molecular and Experimental Medicine, Cardiff University School of Medicine, Cardiff, United Kingdom; 9 Department of Ophthalmology, D-45122 University of Duisburg-Essen, Essen, Germany; 10 Department of Ophthalmology, Johannes Gutenberg University Medical Center, Mainz, 551331 Germany; 11 3rd University Department of Ophthalmology, Aristotle University of Thessaloniki, 1 Kyriakidi Street, 546 36 Thessaloniki, Greece; 12 Department of Endocrinology and Diabetes, Hippokration Hospital of Thessaloniki, Thessaloniki, Greece; 13 Department of Endocrinology and Metabolism, Gazi University, Faculty of Medicine, Besevler, Ankara, 06500 Turkey; 14 Department of Ophthalmology, Fondazione IRCCS Ca’ Granda Ospedale Maggiore Policlinico, Milan, Italy; 15 Department of Ophthalmology, Gazi University Faculty of Medicine, Besevler, Ankara, 06500 Turkey; 16 Faculty of Medicine University of Belgrade, Institute of Ophthalmology Clinical Centre of Serbia, Belgrade, Serbia; 17 Basedow.ch Interdisciplinary Centre for Graves’ Orbitopathy, Fährweg 10, 4600 Olten, Switzerland; 18 School of Medicine, University of Belgrade, Clinic of Endocrinology, Clinical Centre of Serbia, Belgrade, Serbia; 19 Academic Medical Center, 22660 1100 DD Amsterdam, Netherlands; 20 Department of Endocrinology, Level 6, Leazes Wing, Royal Victoria Infirmary, Newcastle upon Tyne, NE1 4LP UK

**Keywords:** Graves’ orbitopathy, Ophthalmopathy, Prevalence, Incidence, Epidemiology, Rare disease, EUGOGO

## Abstract

**Background:**

Graves’ orbitopathy (GO) is an autoimmune condition, which is associated with poor clinical outcomes including impaired quality of life and socio-economic status. Current evidence suggests that the incidence of GO in Europe may be declining, however data on the prevalence of this disease are sparse. Several clinical variants of GO exist, including euthyroid GO, recently listed as a rare disease in Europe (ORPHA466682).

The objective was to estimate the prevalence of GO and its clinical variants in Europe, based on available literature, and to consider whether they may potentially qualify as rare. Recent published data on the incidence of GO and Graves’ hyperthyroidism in Europe were used to estimate the prevalence of GO. The position statement was developed by a series of reviews of drafts and electronic discussions by members of the European Group on Graves’ Orbitopathy. The prevalence of GO in Europe is about 10/10,000 persons. The prevalence of other clinical variants is also low: hypothyroid GO 0.02–1.10/10,000; GO associated with dermopathy 0.15/10,000; GO associated with acropachy 0.03/10,000; asymmetrical GO 1.00–5.00/10,000; unilateral GO 0.50–1.50/10,000.

**Conclusion:**

GO has a prevalence that is clearly above the threshold for rarity in Europe. However, each of its clinical variants have a low prevalence and could potentially qualify for being considered as a rare condition, providing that future research establishes that they have a distinct pathophysiology. EUGOGO considers this area of academic activity a priority.

## Background

Graves’ orbitopathy (GO) (ICD-10 H06.2) [1] is an autoimmune condition that targets the orbits [2] and is responsible for significant morbidity [3, 4] including, in rare cases, blindness [5]. The pathogenesis of GO is closely linked to thyroid autoimmunity [2]. Autoimmune thyroid diseases share a common genetic background, but display widely varying and overlapping clinical phenotypes [6]. Among them, GO is the most striking extra-thyroidal manifestation and the hardest to treat [5, 7]. GO has been described as a rare disease by several authors [8–12], however meaning “uncommon” without a precise definition and to date no direct population surveys of prevalence are available. Rare disease status can raise public awareness and encourage research, leading to improved patient care [13], much of which is needed for patients with GO [14].

The objective of this position statement was to estimate the prevalence of GO and its clinical variants in Europe, based on available literature, and to explore whether rare disease designation may be appropriate.

## Methods

A task force was identified by the officers of the European Group on Graves’ Orbitopathy (EUGOGO). The lead author performed a literature search using PubMed with the key terms “Graves’ orbitopathy”, “Graves’ ophthalmopathy”, “thyroid eye disease” and “prevalence”, “epidemiology”, “incidence”, “rare disease”, “orphan”. Based on the results of the literature search, a brief review of the available evidence and a list of questions relevant to the topic of GO as a rare disease was drafted and circulated to members of the task force for approval. All members of EUGOGO were invited to contribute if they so wished. Topics were allocated to individual authors who were asked to perform additional literature searches and summarise, appraise and comment on the evidence. The submissions from individual members were circulated for review and comment, leading to further discussions, amendments and the final draft.

The prevalence of GO was estimated from publications on the incidence of GO and Graves’ hyperthyroidism (ICD-10, E05.0) [1]. The literature search was limited to studies of patient cohorts derived from European populations that completed recruitment after 2005, in order to capture most recent trends given that the focus of our interest is Europe, and that the epidemiology of GO seems to be changing over time [11, 15–17]. The literature search on the incidence of Graves’ hyperthyroidism yielded 366 publications. Of these, only three studies met the above criteria [18–20]; Table 1.

**Table 1:** Characteristics of studies selected for estimation of prevalence of GO

Study	Study population	Country	Years studied	Type of study
Abraham-Nordling et al. [18]	3.5 million general population	Sweden	2003–2005	Prospective, population registry-based, reporting on incidence of Graves’ hyperthyroidism and all grades of severity of GO
Zaletel et al. [19]	1.0 million general population	Slovenia	1999–2009	Single institution, prospective cohort study of patients with thyroid disease, including Graves’ hyperthyroidism as a subgroup
Laurberg et al. [20]	0.5 million general population	Denmark	1992–2011	Single institution, prospective cohort study, reporting on incidence of moderate-to- severe GO

The prevalence of Graves’ hyperthyroidism, from incidence data, was estimated using established methodologies for calculating global burden of disease [21–23] and the software DisMod II [24]. This is based on a simple model that formalises the relation between incidence (age and sex adjusted), general mortality, case-fatality and prevalence. We assumed that the duration of GO is life-long (though some cases of GO are transient, but difficult to quantify) and that the relative risk of dying due to Graves’ hyperthyroidism is 1.28 (95% CI 1.21-1.36), based on data from Brandt et al. [25]. It was further assumed that within the general population an individual will either acquire GO, or die from a GO-unrelated cause; that patients with GO will either die from a GO-related, or from a GO-unrelated cause; and that the influence of the incidence of a rare disease on the general population is negligible. It follows that the number of subjects within a stable general population at any given moment will depend on the mortality rate and GO incidence, while the number of GO patients will depend on GO incidence, case fatality and general mortality rate. If GO incidence, case fatality and general mortality rate are constant, a set of ordinary differential equations can be defined to characterise movement between general population, diseased, and dead [24].

## Results and discussion

The available data on incidence of GO and Graves’ disease in Europe are limited, but are derived from different geographical areas, are large and internally consistent, and therefore reliable for the purposes of estimating prevalence of GO.

### Calculation of prevalence of GO from incidence of GO

The data provided by one of the major publications on the incidence of GO [20] did not include age-specific intervals. However, the authors stated that “age distribution differed in GO and Graves’ hyperthyroidism, with GO being less than 2% of Graves’ hyperthyroidism at age 20–40 year and 8% at age 40–60 year.” We therefore estimated age and sex-specific incidences derived from [20] and used them to calculate prevalence (Table 2).

**Table 2:** Estimated age and sex-specific incidences derived from Laurberg et al. [20]. These figures were used to calculate prevalence of GO

Age (years)	Incidence of GO (cases/10,000/year)	
	Female	Male
0–20	2.7	0.005
20–40	6.7	0.014
40–60	26.7	0.054
>60	13.4	0.027

This is the only study which directly assessed the incidence of GO in a large European population prospectively and included new cases between 1992 and 2011, based on approximately 8.9 million person-years of observation [20]. The incidence of moderate-to-severe GO was 0.161/10,000/year with a median age at onset of 50 years [20]. Mild cases of GO were not included due to difficulties in defining mild GO in large-scale epidemiological surveys. Distinguishing mild GO from normality and from the transient ocular effects of thyrotoxicosis of any cause, can be a challenge, especially if multiple observers are involved with variable degrees of expertise. In recent cross-sectional studies from secondary or tertiary centres, conducted by highly trained observers and with well-defined criteria for GO, about 65% of all cases of GO were found to have mild GO, and about 2% sight-threatening, disease [11, 16]. We assumed that the cases of mild GO which were excluded by Laurberg et al. [20], accounted for 2/3 (65%) of all cases of GO. Hence, the adjusted figure for incidence of all grades of severity of GO, based on the above studies [11, 16, 20], is 0.483/10,000/year (incidence of moderate-to-severe disease 0.161 multiplied by 3, in view of the fact that it represents 1/3 of all cases = 0.483). The data published by Laurberg et al. [20] are unclear as to whether sight-threatening GO was included within the moderate-to-severe category. We therefore used the frequency of sight-threatening GO of 2% cited by other publications [11, 16] to calculate the prevalence of sight-threatening GO from data by [20]. Using the approach described in “methods” the prevalence of GO is estimated to be 8.97/10,000 population. It can be further broken down to mild (5.83/10,000), moderate-to-severe (2.96/10,000) and sight-threatening GO (0.18/10,000) (Table 3a).

**Table 3:** Estimated prevalence of GO and variants of GO. (a) shows prevalence by severity and (b) for clinical variants (all grades of severity)

	PREVALENCE (per 10,000 population)	PROPORTION OF PATIENTS WITH VARIANT	SOURCE
*(a)*			
All cases of GO	8.97	-	[18]
	15.48		[19, 20]
Mild GO	5.83	65.0%	[18]
	11.03	72.8%	[19, 20]
Moderate-to-severe	2.96–4.45	33.0–29.4%	[18–20]
Sight-threatening	0.18	2.0%	[11, 14]
*(b)*			
Euthyroid/hypothyroid GO	0.02–1.10	0.2–11.0%	[29–33]
GO associated with dermopathy	0.15	1.5%	[7]
GO associated with acropachy	0.03	0.3%	[7]
Asymmetrical GO	1.00–5.00	10.0–50.0%	[34–36]
Unilateral GO	0.50–1.50	5.0–15.0%	[35–38]

### Calculation of prevalence of GO from incidence of Graves’ hyperthyroidism

Based on data for incidence of Graves’ hyperthyroidism, the calculated incidence of GO in Europe has been reported to be 10–30/10,000/year [26–28]. These studies relate to epidemiological data collected 10–20 years ago and the estimates may not be valid today since the incidence of GO appears to be declining [11, 14, 15] although this may be partly due changing referral patterns. In a recent study, Abraham-Nordling et al. [18] reported the incidence of Graves’ hyperthyroidism derived from a Swedish population of about 3.5 million. The incidence of Graves’ hyperthyroidism was 2.10/10,000/year. Mild (“non-infiltrative”) GO was present in 15.2% of patients with Graves’ hyperthyroidism and moderate-to-severe/sight-threatening (infiltrative) in 4.9% of cases. Thus, the incidence of all cases of GO was 0.42/10,000/year (incidence of mild GO 0.32/10,000/year and of moderate-to-severe/sight-threatening 0.10/10,000/year) [18]. Using the same methodology for calculating prevalence as before, the prevalence of GO is shown in Table 3a. The study by Zaletel et al. [19] yielded remarkably similar results for the incidence of Graves’ hyperthyroidism as that by Abraham-Nordling et al. [18] (2.08/10,000/year), and identical figures for the prevalence of GO (Table 3a).

The figures for overall prevalence of GO derived from [18] and [20] vary almost by a factor of 2. The reason is unclear but it may represent a true variation in different populations, or may be accounted for by the different methodologies in identifying cases. Laurberg et al. [20] presumed that all patients with moderate-to-severe GO were seen in a single tertiary centre. Hence, the data have included some patients with GO seen elsewhere and patients who were not referred. The other study [18] identified patients with Graves’ hyperthyroidism through a registry and it may have captured more cases. One of the weaknesses of our calculations is that the estimates of prevalence of GO are derived from a small (*n* = 3) number of epidemiological studies. However, this is all that is available in the recent literature and it is unlikely that more extensive data will be forthcoming for a while. The consistency between the figures from the three studies quoted above is sufficient in our view to justify estimates of prevalence based on these data.

### Prevalence of variants of GO

A number of distinct clinical variants of GO exist, however these are poorly studied and deserve attention. Euthyroid/hypothyroid GO is a variant comprising of patients who are euthyroid, or have overt or subclinical hypothyroidism, before or within 6–12 months after the onset of GO and constitutes 0.2–11% of all cases of GO [29–33]. Patients with GO associated with dermopathy have characteristic skin changes and comprise 1.5% of all cases of GO [7]. Patients with GO associated with acropachy display typical nail and subperiosteal changes and make up 0.3% of all cases of GO [7]. Asymmetrical GO, usually defined as a difference of ≥2 mm in proptosis between the two eyes, is reported to occur in 10–50% of patients with GO [30, 34–36]. Unilateral GO, defined as one of more features of GO in one eye without any in the contralateral eye, is reported to occur with a frequency of 5–15% of all cases of GO [35–38]. Previously reported data on the frequency of euthyroid and asymmetrical/unilateral GO are likely to be overestimates as awareness of IgG4-related ophthalmic disease has been low [39, 40], but is impossible to quantify. The estimated prevalence of these variants (inclusive of all grades of severity) based on the previously estimated overall prevalence of GO of approximately 10/10,000 population, and the reported frequencies of the variants is shown in Table 3b.

One of our assumptions in calculating the prevalence of GO was that the duration of GO is life-long. In a minority of patients this is not the case. In a study published 20 years ago by Bartley et al. [41], 40% of patients responding by questionnaire reported that their eyes were normal 10 years after the diagnosis of GO, but this was not possible to confirm objectively. A study published in 2002 by Terwee and colleagues [42] reported on outcomes in a cohort of patients after 11.7 years of follow-up, but the data did not report on the frequency of complete regression of the features of GO. In a more recent study a significant proportion of patients had transient GO [11]. However, this was a selected group of patients with Graves’ hyperthyroidism who had no GO at the time of recruitment and were treated with anti-thyroid drugs, from a single institution. It is therefore impossible to correct for transient GO with an acceptable degree of confidence without taking a risk to over-state the case for variants of GO being rare. However, it is important to highlight that the data in Table 3 are likely to be overestimates of the prevalence of GO.

Designation of rare disease in Europe requires that several conditions be satisfied: prevalence of less than 5 per 10,000 population [43], distinct phenotype, distinct aetiology and mechanism of pathogenesis [44] and seriously debilitating or life-threatening impact on patients [45]. Euthyroid GO achieved rare disease status on the basis of the above criteria, and the consideration that the euthyroid status of such patients reflects mechanistically unique processes that are distinct from Graves’ hyperthyroidism. We speculate that the other clinical variants described above may also have underlying unique and distinct pathogenetic processes from Graves’ hyperthyroidism and each other. Future research focusing on deep phenotyping and molecular characteristics of variants of GO is likely to be productive in understanding the pathogenesis of GO.

## Conclusions

GO has a prevalence that is clearly above the threshold for rarity in Europe. However, each of its clinical variants have a low prevalence and could potentially qualify for being considered as a rare condition, providing that future research establishes that they have a distinct pathophysiology. EUGOGO considers this area of academic activity a priority.

## Abbreviations

EUGOGO: European Group on Graves’ Orbitopathy
GO: Graves’ orbitopathy
